# Data science approaches to infectious disease surveillance

**DOI:** 10.1098/rsta.2021.0115

**Published:** 2022-01-10

**Authors:** Qingpeng Zhang

**Affiliations:** School of Data Science, City University of Hong Kong, Hong Kong

**Keywords:** infectious disease, mathematical modelling, data science, big data, COVID-19

## Abstract

Novel data science approaches are needed to confront large-scale infectious disease epidemics such as COVID-19, human immunodeficiency viruses, African swine flu and Ebola. Human beings are now equipped with richer data and more advanced data analytics methodologies, many of which have become available only in the last decade. The theme issue *Data Science Approaches to Infectious Diseases Surveillance* reports the latest interdisciplinary research on developing novel data science methodologies to capitalize on the rich ‘big data’ of human behaviours to confront infectious diseases, with a particular focus on combating the ongoing COVID-19 pandemic. Compared to conventional public health research, articles in this issue present innovative data science approaches that were not possible without the growing human behaviour data and the recent advances in information and communications technology. This issue has 12 research papers and one review paper from a strong lineup of contributors from multiple disciplines, including data science, computer science, computational social sciences, applied maths, statistics, physics and public health. This introductory article provides a brief overview of the issue and discusses the future of this emerging field.

This article is part of the theme issue ‘Data science approaches to infectious disease surveillance’.

Infectious disease epidemics have generated massive data on human behaviours, including human movement, contact tracing, clinical records, virology, pharmacy, scientific literature and so on. With the ever-increasing availability of data and the urgent need for data-driven insights to combat various infectious disease epidemics, notably the ongoing COVID-19 pandemic, data science approaches have emerged to become widely adopted in understanding and combating infectious disease surveillance [1].

This highly selective theme issue of *Philosophical Transactions A* features frontline scholars worldwide to present their latest data science research on combating the COVID-19 pandemic and other infectious disease epidemics, including human immunodeficiency viruses (HIV), diarrhoeal and respiratory disease. The lineup of contributors features both early/mid-career and well-established scholars in data science, computer science, computational social sciences, applied maths, statistics, physics and public health. The choice of 12 research articles spans from uncertainty quantification and epidemiological parameter estimation to modelling human mobility using mobile phone data and fundamental issues of the prediction of the epidemic. One review paper summarizes the newly-born data science approaches to confronting the COVID-19 pandemic. This issue aims to ignite new research on data science approaches to infectious disease surveillance, modelling and control.

Most infectious diseases are contagious in humans by close contact. Therefore, the outbreak and progression of epidemics are heavily dependent on local and international human mobility. Recent advances in information and communications technology and the wide adoption of smart devices enable access to rich human mobility data. Three papers focus on modelling human mobility for infectious disease monitoring and control. Jianxi Gao and colleagues harnessed large-scale crowd-sourced human mobility data to construct the temporal (daily) hierarchical networks depicting human movements within and between Metropolitan Statistical Areas in the USA [2]. They examined the percolation effect on these temporal hierarchical networks and revealed the presence of functional sub-units with high mobility thresholds. These data-driven insights facilitate our understanding of the temporal community structure of mobility networks during travel restrictions and non-pharmaceutical interventions (NPIs).

In another study in Denmark [3], Laura Alessandretti and colleagues characterized how different travel behaviours contribute to the spread of infectious diseases by using non-negative matrix factorization. They found that the Danish mobility patterns could be decomposed to the travels during workdays, weekends and holidays. They calculated the effective distance between municipalities to examine how each type of mobility contributes to the spread of COVID-19. These findings shed light on the effect of travel restrictions on different aspects of people’s travel behaviour during the pandemic and could potentially inform effective policies for mitigating the pandemic and other epidemics.

Human mobility also plays a crucial role in the spread of HIV among at-risk populations, such as men who have sex with men (MSM). Dan Wu and colleagues characterized the behavioural dynamics underlying the spread of HIV within and between cities in Guangdong province, China, by harnessing the multi-sourced big data from MSM social networking sites, offline human mobility network, and self-reported sexual behaviours among MSM [4]. Their study demonstrated that the adoption of pre-exposure prophylaxis (PrEP) by MSM significantly delayed the occurrence of HIV in all cities. Results also showed that hubs in the human mobility network had a higher risk of early exposure to new HIV genotypes. These findings verify the efficacy of PrEP adoption among MSM and provide the data-driven evidence to prioritize cities for the control and prevention of HIV in Guangdong province.

The heterogeneity in the susceptibility and contact intensity highlights the need to examine the age-specific severity of COVID-19. Alex Arenas and colleagues found that the proportion of elderly infections was very small during low-prevalence periods and increased during the high-prevalence periods in eight countries or regions [5]. The authors proposed a mechanistic explanation for this phenomenology by accounting for the age-specific severity of COVID-19, and modelled the dynamic adoption of heterogeneous NPIs through a two-strategy game in an susceptible-infectious-removed (SIR) transmission model. Their results provided insights into the understanding of the temporally varying case distribution among different age groups, and had important implications in how minimal models can exhibit the complex phenomenology in real data. In practice, the results highlighted the need to account for the underestimation of future stress on the healthcare system because of the low fraction of infections among the at-risk group.

Age-specific mixing patterns may also affect the effectiveness of several NPIs, such as school closure, a common control measure deployed worldwide to control COVID-19. By harnessing the surveillance data from two Chinese cities, Joseph T. Wu and colleagues inferred the susceptibility of people in different age groups, and developed transmission models to assess the impact of school closure in reducing the spread of COVID-19 [6]. They found that the low susceptibility of school children limited the effectiveness of school closure in COVID-19 control and suggested that school closure may not be the ideal primary intervention for controlling COVID-19.

Heterogeneity also lies in the growth rate of the disease at geographical scales. Kristina Lerman and colleagues addressed this critical problem by analysing confirmed infections and deaths over multiple geographic scales in the United States [7]. They found that the impact of COVID-19 is highly unequal across regions. A Reed-Hughes-like mechanism was proposed to model such effects. The results highlighted the trade-off between the lowered noise and increased bias by spatial aggregation and called for the attention of public policymakers to address this aggregation distortion in estimating the growth rates of COVID-19.

Most of the COVID-19 transmission models were based on the standard SIR compartmental model or its variants. Three papers discussed the calibration of such compartmental models and their limitations in handling real data on this issue. James Gleeson and colleagues reported the population-based SEIR (E stands for Exposed) model that advised the Irish government on COVID-19 responses [8]. To capture the effect of NPIs, their model has a time-varying effective contact rate. They proposed a novel algorithm for robust calibration using observed data. Their calibration algorithm can be applied to the modelling of other scenarios (e.g. vaccination programs) with good accuracy at lower complexity.

In [9], Dirk Helbing and colleagues pointed out the fundamental challenges in using SIR-type compartmental models to monitor epidemics because of the neglect of stochastic and network effects, and the role of the measurement process. They combined such compartmental models with a measurement model of the testing process and examined the errors and biased sampling. They concluded that applying a conventional approach to complex systems with nonlinear dynamics, network effects and uncertainty could be misleading, putting the downstream monitoring and forecasting tasks at stake. They argued that these errors could be corrected by incorporating the scientific knowledge of the spreading dynamics and the measurement process into the models.

Another apparent limitation of SIR-type compartmental models is the simplified assumption of uniform and fully mixed population in which social networks are formed. To characterize the heterogeneity in disease transmission among individuals, Nicholas A. Christakis and colleagues collected detailed longitudinal sociocentric data in Honduras and created a social-network-powered transmission model to identify super-spreaders and the vulnerable individuals in the population [10]. Through agent-based simulations, they were able to predict the outbreaks of diarrhoeal and respiratory disease. Population-level surveys were used to validate the predictions and the identification of super-spreaders and vulnerable individuals. Different from retrospective contact tracing, in this study, super-spreaders were proactively identified by simulations. Their model can be applied to other contact-dependent communicable infectious diseases, and the results strengthen the need to consider the social interactions in disease transmission models.

A major task in infectious disease surveillance is to obtain an accurate estimate of the disease prevalence via testing, which is challenging because of the errors and biases in testing in dynamically evolving populations. Lucas Böttcher and colleagues developed a statistical model for inferring the disease prevalence [11]. Their model allowed for biases in sampling and errors in testing. Validation with actual COVID-19 testing data demonstrated the effectiveness of the proposed model in estimating the disease prevalence with uncertainty quantification of indices. The proposed modelling framework is generic and can be easily applied to the surveillance of other infectious diseases.

Social media has been recognized as a major data source for the surveillance of infectious diseases and public health events. Wei Wang and colleagues developed the COVID-19 Surveiller, a web-based COVID-19 surveillance system [12]. COVID-19 Surveiller features a dynamic graph neural network model to forecast the trends and identify the high-risk events of COVID-19 by analysing streaming Twitter data.

Resource allocation is critical in infectious disease control. By harnessing the real-world implementation data from Chinese MSM, Weiming Tang and colleagues formulate two data-driven integer linear programming models to optimize the secondary distribution of HIV self-testing (HIVST) kids among high-risk populations [13]. Results demonstrated the feasibility of the proposed data-driven approach in improving the health economic benefit of HIVST secondary distribution. These models could be used as a reference to guide the implementation of secondary HIVST distribution in low- and middle-income countries, where resources are scarce. Further quasi-experimental trials will be conducted to compare the actual economic benefits of the proposed methods with that of traditional public health approaches.

There are a number of important topics that were not covered by the papers in this theme issue, including assessing the economic impact, mining patients' data, drug re-purposing and development, mining scientific literature and so on. Interested readers can refer to [1], which reviews the newly-born data science approaches to addressing these problems in the battle against COVID-19, and discusses the opportunities and challenges in this emerging field.

It has become evident that big data and data science approaches are indispensable for the effective control of infectious diseases. There was always a gap between conventional public health and data science researchers. We hope that this theme issue can bridge this gap by featuring contributions from experts in both fields. Looking forward, the research reported in this issue not only contributes to combating the COVID-19 pandemic but also the surveillance and control of other infectious diseases.

## Data accessibility

This article has no additional data.
